# Human Thelaziosis Caused by *Thelazia callipaeda* Eyeworm, Hungary

**DOI:** 10.3201/eid2812.220757

**Published:** 2022-12

**Authors:** Hajnalka Juhász, Géza Thury, Mária Szécsényi, Edit Tóth-Molnár, Katalin Burián, Zoltán Deim, Gabriella Terhes

**Affiliations:** University of Szeged, Szeged, Hungary

**Keywords:** thelaziosis, nematodes, parasites, Europe, human infection, vector-borne infections, Hungary

## Abstract

Ocular infections with *Thelazia callipaeda* eyeworms in Europe have become more common. We report a case in Hungary caused by *T. callipaeda* eyeworms in a 45-year-old woman who had no travel history abroad.

*Thelazia* spp. (Spirurida*,* Thelaziidae) are vectorborne zoonotic nematodes that can parasitize conjunctiva and surrounding structures of wild and domestic animals as well as humans (*1*). Before 2022, a total of 16 species of *Thelazia* had been described; 3 species, *T. callipaeda*, *T. californiensis*, and *T. gulosa*, are known to infect humans. *T. callipaeda* nematodes, commonly known as eyeworms, cause autochthonous cases in Europe (*2*). The earliest reported endemic infection in Europe was detected in a dog in the Piedmont region of Italy in 1989. Since then, several animal and human cases have been documented throughout Europe (Appendix Table 1) (*1*–*4*). In Europe, under natural conditions, the only known vector and intermediate host of *T. callipaeda* eyeworms is the lachryphagous male *Phortica variegate* fly *(**1**,**5**)*. The biologic activity of the fly is affected by temperature (20°C–25°C) and relative humidity (50%–75%) (*1*,*6*). The most common clinical manifestations of *T. callipaeda* infections are lacrimation, foreign body sensation, itchiness, conjunctivitis, and follicular hypertrophy of the conjunctiva; the affected eye may also show severe keratitis and corneal ulceration. Treatment of this infection in humans is primarily the mechanical removal of worms, which is more difficult in their immature stages (*7*).

In Hungary, *T. callipaeda* infection has been described in dogs (*3*). We report a case of conjunctivitis in a human caused by *T. callipaeda* eyeworms. Our goal is to draw the scientific community’s attention to this spillover event.

A 45-year-old woman in Hungary was referred to an ophthalmologist in September 2020; she had a foreign-body sensation and redness in her left eye for a week. Slit-lamp examination revealed conjunctivitis. Empiric tobramycin and dexamethasone therapies were initiated. At the time of follow-up, 3 thin, creamy white live worms were removed from the conjunctival fornices of her left eye (Figure). Left eye examination revealed follicular conjunctival hypertrophy. The cornea was not affected, visual acuity was 20/20 on both eyes, and intraocular pressure was in the normal range. The patient’s medical history was uneventful. Laboratory examination showed no elevated leukocytes, C-reactive protein, or erythrocyte sedimentation rate. The patient had no peripheral blood eosinophilia. Because of worsened conjunctivitis, 2% boric acid was applied for 5 days after removal of the worms. On the last follow-up visit, the patient had no symptoms.

**Figure:**
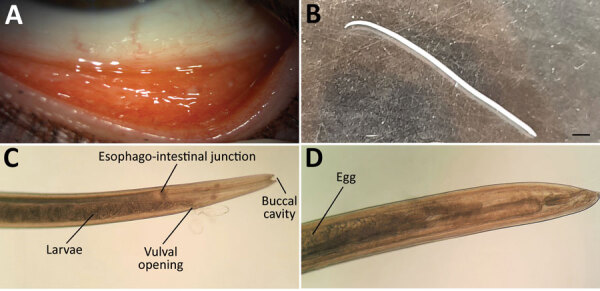
Imaging results for *Thelazia callipaeda* eyeworm infection in a woman in Hungary. A) Follicles in the inferior tarsal conjunctiva in the patient’s left eye 5 days after removal of an adult female *T. callipaeda* worm. B) Female worm removed from the patient’s left eye. Scale bar indicates 1 mm. C, D) Morphologic characteristics of the *T. callipaeda* worm from the patient (C) and eggs visible within the specimen (D). Original magnification ×100.

Two worms were sent to the Department of Medical Microbiology, University of Szeged (Szeged, Hungary). We examined them under a light microscope (Leica DM 100; Leica Microsystems, https://www.leica-microsystems.com). Wet-mount preparation showed the presence of a characteristic vase-shaped buccal cavity (Figure, panel C) and serrated cuticula with transverse striations. The position of the vulva was anterior to the esophago-junction (*8*). The anterior half of the abdominal cavity contained first-stage larvae; the posterior part contained eggs. The nematodes were 15 mm and 14 mm long. We identified them as female *T. callipaeda* eyeworms on the basis of morphologic features. We isolated total DNA from one using QIAamp DNA mini kit (QIAGEN, https://www.qiagen.com) in accordance with tissue protocol; we then performed an in-house PCR targeting *cox*1 as described previously by Čabanová et al (*9*). Sequence analysis of the amplified PCR product (GenBank accession no. OP278871) showed 100% homology with *T. callipaeda* haplotype 1 strains in GenBank (Appendix Figure 1).

We describe an autochthonous case of a patient with *T. callipaeda* eyeworm ocular infection in Hungary. The patient had no travel history abroad. In July, she visited the Bükk National Park in northeastern Hungary, where she saw a lot of flies. This vector is widely distributed in southern and central Europe and exists in Hungary as well (*1*,*10*). Its lachryphagous activity depends mostly on temperature, so climatic changes affect the spread of infection toward the north, affecting new areas (*1*,*6*). Until December 2017, a total of 10 canine thelaziosis cases were identified by Farkas et al. in Hungary (*3*). Most of them visited the same park located in Borsod-Abaúj-Zemplén as our patient (*3*). That study also suggested that wild carnivores, mainly red foxes, had a role in spreading thelaziosis beyond the border (*3*). The emergence of human thelaziosis may be explained by the fact that the number of red foxes in Hungary has tripled during the past 50 years (Appendix Figure 2) (Appendix reference *11*). In human case-patients, the first-choice therapy is to remove the worms mechanically by flushing the conjunctival sac with sterile physiologic saline under local anesthesia (Appendix reference *12*).

From a therapeutic and epidemiologic standpoint, it is important to differentiate between infectious and allergic conjunctivitis. Furthermore, diagnosis can be difficult because immature larvae can hide in the excretory ducts of the lacrimal glands (*7*). Our findings indicate the need for education and raised awareness about this infection especially for ophthalmologists. Early and adequate diagnosis can help to prevent complications such as corneal ulceration.

Appendix Additional information about human thelaziosis caused by *Thelazia callipaeda* eyeworm, Hungary.
