# Supplementary material for: Human Thelaziosis Caused by Thelazia callipaeda Eyeworm, Hungary
Source: Emerg Infect Dis. 2022 Dec;28(12):2559–61. doi: 10.3201/eid2812.220757 (PMC9707569; doi:10.3201/eid2812.220757)
Supplement: Appendix — Additional information about human thelaziosis caused by Thelazia callipaeda eyeworm, Hungary. [file 22-0757-Techapp-s1.pdf]

Article DOI: https://doi.org/10.3201/eid2812.220757

Human Thelaziosis Caused by *Thelazia callipaeda* Eyeworm, Hungary

Appendix

Appendix References

11. Csányi S, Márton M, Major FC, Schally G. Hungarian game management database. 2020/2021 hunting year. Gödöllő, Hungary: National Wildlife Management Repository; 2021.

12. Shen J, Gasser RB, Chu D, Wang Z, Yuan X, Cantacessi C, et al. Human thelaziosis—a neglected parasitic disease of the eye. J Parasitol. 2006;92:872–6. PubMed https://doi.org/10.1645/GE-823R.1

Appendix Table. Reports of animal and human cases of *Thelazia callipaeda* infection from Europe*

Country	Animal cases	Human cases
Italy	x	x
France	x	x
Switzerland	x	
Germany	x	x
Spain	x	x
Portugal	x	x
Belgium	x	
Bosnia and Herzegovina	x	
Croatia	x	x
Serbia	x	x
Romania	x	
Greece	x	
Bulgaria	x	
Slovakia	x	
United Kingdom	x	
Turkey	x	
Austria	x	
Hungary	x	
Czech Republic	x	
Moldova	x	

*Sources: Palfreyman et al. (1); do Vale et al., (2); Farkas et al. (3); Morgado et al. (4)

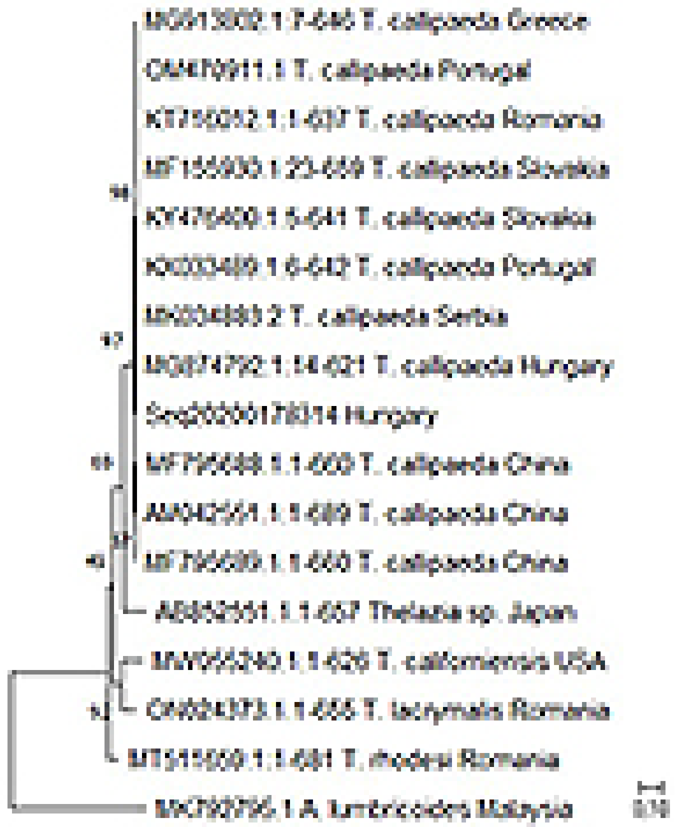

Appendix Figure 1. Maximum-likelihood tree shows the relationships between *cox*1 sequences in the case of our isolate (Seq20200178314) and other *Thelazia* spp. retrieved from GenBank. *A. lumbricoides* is designated as an outgroup.

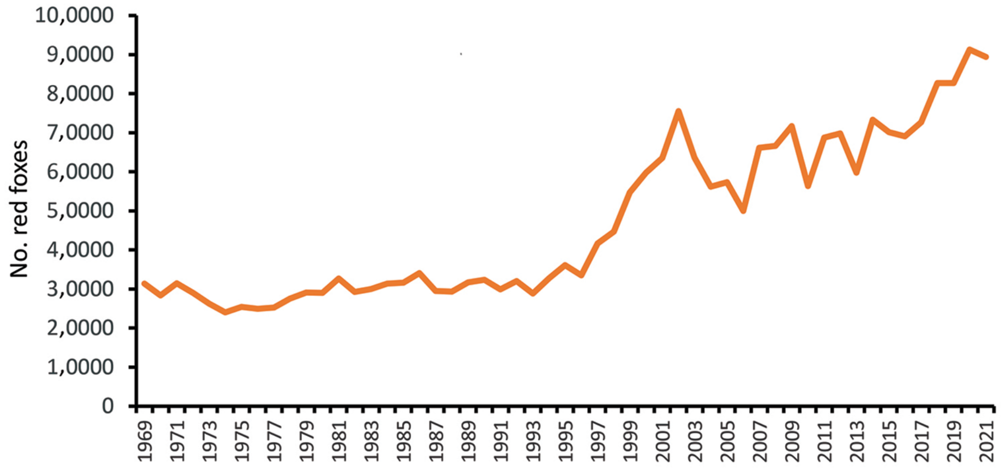

Appendix Figure 2. Changes in the number of red foxes (*Vulpes vulpes*) in Hungary during the last 50 years.
